# Medical Opinion and Movement

**Published:** 1910-12-17

**Authors:** 


### Medical Opinion and Movement.

### Menopause in the Male Sex.

In a contribution to the *Centralblatt für Neurologie* Dr. K. Mendel draws a clinical picture of a group of symptoms, chiefly of a subjective nervous order, which he thinks occur more frequently than is generally supposed in men sometime about the age of fifty years, and which he attributes to physical changes in the organism similar to those taking place at the menopause in women. The affection starts between forty-seven and fifty-seven, more usually between fifty and fifty-four. The patients have frequently enjoyed good health up to that time and have shown no evidence of neurosis. They complain of feelings of discomfort and anxiety, they feel weak and develop an excessive sensitiveness, which makes them easily give way to tears. They suffer from congestive headaches, palpitation, sudden sweats, insomnia, vertigo, and a variety of aches and pains and paræsthesiæ. There is often a deficiency in memory, especially for recent events, they become indifferent, morose, and spend their time in ruminating over pessimistic ideas in regard to themselves or their family. They lose pleasure in life and may contemplate suicide. Sexual desire is diminished. Clinical examination reveals no objective sign. The condition usually clears up in the course of six months to four years. Such is the picture drawn by Dr. Mendel, and he points out that it may be difficult to distinguish the condition from the psychic phenomena of arterio-sclerosis, which are, however, accompanied by certain definite objective signs, or from ordinary neurasthenia or hypochondriasis. The author points out that the symptoms are closely similar to those of the menopause in women, and are probably due to a like cause—a diminution in the secretion of the genital glands. He suggests that the condition is not so frequently observed in men, because many cases of a mild character would not reach the physician, and, further, in many men suppression of the sexual functions does not occur till an advanced age. For treatment of the condition the author advises the needle baths and saline baths with carbonic acid, and the administration of bromides, sometimes combined with iodides and opium, and, above all, psychotherapy.

### Constipation and Toxæmia.

In recent years, owing chiefly to the great advances in bacteriological knowledge, the theory has gradually gained ground among the medical profession that a great deal of general ill-health and a large number of chronic affections, as well as acute infections, are due to prolonged toxæmia caused by the absorption of toxins resulting from the activity of micro-organisms in some part of the system. In consequence much more energetic measures are taken to combat any sort of continual chronic inflammatory condition of the mucous membranes and the like than heretofore, such as nasal catarrh, uterine discharges, and so on. A good deal of ill-health, for instance, has been traced to carious teeth and an unhealthy condition of the mouth, and a large number of gastric conditions, conditions of anæmia, and so forth, have been cured by proper dental administration. More recently attention has been directed to the toxæmia that may result from absorption of toxins from an unhealthy and stagnant bowel, or what is characterised by Mr. Arbuthnot Lane as "intestinal stasis." The operation of short-circuiting the lower bowel, which this surgeon has made peculiarly his own, appears to have given good results in severe cases, but there are a large number of cases of a much milder degree in which operative interference of this nature would be deemed undesirable and unjustifiable.

For such cases great hopes were entertained for the treatment with sour milk, and although some good appears to have been derived from the treatment in certain cases, the results have been on the whole disappointing. What would appear to be required is some inert substance which will lubricate the bowel contents in a similar way to the natural mucous secretion without setting up any excessive stimulating effect, as in the case of ordinary aperients. Such a substance we seem to possess in liquid paraffin or vaseline, and the daily administration of this substance has already been attended with satisfactory results in a large number of cases. Two or three teaspoonfuls to a tablespoonful of liquid paraffin or vaseline are given two or three times a day. Although the purified paraffin is quite tasteless, its oleaginous nature makes it very distasteful to many, and the problem presents itself to the pharmacist how such a preparation can be made palatable. The question is now being energetically pursued at Guy's Hospital. It is possible that of the two rival substances, vaseline and liquid paraffin, vaseline will eventually be found the most suitable to dispense in a palatable form by combining it with some gritty finely-divided powder, and flavouring with some tart astringent, such as red-currant juice or lemon.

### Ovarian Carcinoma.

In a most instructive paper on metastatic carcinoma of the ovaries, read to the Medical Society of London and published in the *Clinical Journal*, Mr. Bland Sutton announces that after a quarter of a century of study he is still doubtful of the occurrence of primary ovarian cancer. In every case of that neoplasm in that region he has found a primary focus elsewhere, and microscopic examination of the ovarian growth has always shown the structure characteristic of the organ in which the growth originated. In other words, ovarian cancer is frequent as a secondary metastatic process, but so rare as a primary phenomenon that in all Mr. Sutton's enormous experience he has never yet met with it. The primary tumour may be small as compared with the secondary one, and careful search may be necessary to reveal it. Even in those not very rare cases in which a large cystic ovary is found to contain a portion of solid substance which proves to be carcinomatous in structure, Mr. Sutton has always found a primary focus; he believes that in such patients a simple cystic tumour has existed in a woman who develops elsewhere a malignant growth; and that by permeation the cyst has become infected with cells from the latter, so that a mixed mass of cyst and carcinoma results. The Fallopian tube is a source of the malignant process in quite a number of cases, and it is not difficult to see how easy such permeation and infection must be when that is the source of the mischief.

### Stimulation of Bony Growth Artificially.

It appears from the researches of Dr. Meisenbach, published in the *American Journal of Orthopedic Surgery* and commented on by the *Medical Record*, that by taking thought man may shortly be able to add cubits, not merely to his stature but to the length of any individual bone he chooses. His experiments have been conducted upon growing rabbits, and consisted of endeavours to stimulate bony growth by influencing the cellular structures of the epiphysial cartilages by chemical and mechanical means. Into the right tibia just below the epiphysial line he deposited various substances, and carefully studied the effects. Among the irritants used were sterile water, sterile graphite, vaccine of staphylococcus pyogenes aurens, vaccine and graphite, tincture of iodine, pure carbolic acid, alcohol (95 per cent.), and formalin. The latter produced the best results, as far as bone formation is concerned. Acting as both a chemical and (owing to its insolubility) a mechanical agent, the effect is local and not systemic. It is said to cause the formation of osteogenetic tissue by increasing the number of osteoblasts derived from the perichondrium. The stimulation of bony growth in the animals used was observed by comparing the injected limb with that of the opposite side; and it seems definitely proved that even by a single injection it is possible to make any given epiphysis produce more bone than it would otherwise do. The clinical applications of this fact, as applied to shortening of limbs due to fractures, tuberculosis, osteomyelitis, and other causes, appear to be well worth further research.

### Atony of the Bladder.

There are certain cases of atony of the bladder which Mr. J. W. Thomson Walker classifies as apart from the two great types of this condition as seen respectively in obstruction to the outflow of the bladder and in organic diseases of the nervous system. He describes in the *Annals of Surgery* twelve cases of this condition, which has not hitherto received the attention which Mr. Walker thinks that it deserves. The atony is of varying degree; the urethra shows no signs of obstruction, and the prostate is healthy; there are no signs of organic nervous disease. In a number of cases the atony has been present for a sufficient period of time to be certain that tabes or other spinal disease will not develop. There are acute and chronic cases. As regards ætiology, there is little to say; two of the twelve patients admitted syphilis, four had had gonorrhœa, and one had had plumbism. A few similar cases are recorded in the literature, but only a few. The common features of the cases are the gradual onset and increase of difficulty of micturition. The flow does not start promptly; there is a pause of some seconds or minutes before the urine begins to pass. The stream is feeble; it may begin with fair force and then fall away to a dribble, or it may dribble at the start, increase gradually in strength, and then relapse again. The stream is often intermittent, running only while the diaphragm is descending. Chronic distension of the bladder is fairly frequent; the normal desire to micturate as the bladder distends is often lost.

### The Metabolism of Healthy Babies.

The metabolic processes of the sick child have been exhaustively studied in recent years in Germany and France; but not nearly so much work has been done on the infant in health. Hoobler records in the *Archives of Pediatrics* an extensive series of observations on a healthy infant, fed on suitably diluted cow's milk of varying composition. The research was mainly concerned with the nitrogenous metabolism. Nitrogen, he finds, is very well absorbed by healthy infants thus artificially fed; the percentage absorbed varies but little from day to day, and is much the same at varying ages, even though the intake of nitrogen per kilo differs greatly. Nitrogen absorption is carried on equally well under low, medium and high percentages of fat in the milk mixture. The proportion of the food nitrogen which is retained is about one-third. Retention takes place in much the same percentage whether there is a low, medium, or high percentage of fat; and also whether there is a low, medium or high percentage of proteid. In high proteid feeding there is a large amount of nitrogen absorbed and eliminated, thus causing more work to be performed by the infant than on a lower proteid feeding scale. Body weight may diminish notwithstanding a fair nitrogen retention. Elimination is chiefly by the urine; considerably more than half the nitrogen ingested leaves the body thus. A very small quantity is found in the fæces. The author warns his readers against taking these conclusions as absolutely definite, because they are based on the examination of only one child.

### Emaciation Due to Nervous Shock.

An extraordinary case, treated successfully with an extraordinary remedy, is that communicated by Dr. E. Holländer to the *Münchener Mediz. Wochenschrift*. His patient suffered a severe shock by seeing her father brought home dead as the result of an accident. Thereafter she emaciated steadily and progressively until her repulsive appearance prevented her from earning her living. Curiously, this affected her only down to the hips; thence downwards she remained plump, and even put on fat. Above the waist her skin hung in dry yellow folds, so that she resembled a mummy or a corpse. It should be added that no organic disease of the nervous system could be detected. Numerous remedies had been tried without success (including thyroid extract), when the author decided to try injections of a mixture of human and mutton fat. This he has injected subcutaneously, as a substitute for oral feeding, for some years in cases where the stomach or intestines have been subjected to operative measures. He uses removed lipomata and epiploceles, cuts them up fine, and removes as much of the stroma as possible. Then the fat is boiled for three hours in a water-bath, after which it is decanted into sterile jugs and hermetically sealed until wanted for use. As thus obtained human fat is liquid, but if mutton fat be mixed with it a semi-solid mass is obtained which can be melted easily and injected (like paraffin) through a warmed syringe. It then forms a uniform soft mass under the skin, some of which is absorbed; the rest becomes infiltrated by the cells of the part, and a satisfactory cosmetic result ensues. In this particular case the woman was changed from a repulsive hag to a rosy-faced young woman, and was able to regain a position on the stage which she had previously lost.

### The Galactagogue Action of Infundibulin.

In the *Monthly Cyclopædia and Medical Bulletin* Drs. Isaac Ott and J. C. Scott deal briefly with this topic. They find in the goat that in the early puerperium infundibulin injected into a vein in the ear rapidly and greatly increases the flow of milk. They inserted a cannula into the nipple, and by a water aspirator produced the suction necessary to empty the udder. The milk thus aspirated before and after the injection of infundibulin was caught in a graduated flask and measured every five minutes. The increased flow of milk is not due to an increased flow of blood to the udder, because infundibulin contracts the arterioles. These observations can be correlated with the increased size of the pituitary body during pregnancy, although it is true that in these cases the enlargement is chiefly in the anterior lobe. Whether this galactagogue action can be made useful clinically remains to be seen.

### Tuberculosis in France.

Dr. Robin, at a recent meeting of the Paris Academy of Médecine, protested against the statement that 150,000 deaths occur annually in France from pulmonary tuberculosis. These figures are quite inaccurate, and the report published by the Minister of the Interior shows that in 1900 the actual number of deaths from phthisis was 85,271, or 21.7 per 1,000 of inhabitants. It has been objected that many deaths from phthisis have been included under different categories, such as acute bronchitis; but this statement is not supported by facts, for the numbers of deaths from these other diseases have shown a marked diminution of late. It is, therefore, obvious that of recent years the anti-tuberculous campaign carried out with such energy in France is commencing to have an effect on the health of the country as a whole. Measures used have for the most part been preventive rather than curative. The sanatorium is in far less vogue there than in other countries; but the author declines to express a definite opinion as to its merits. He maintains that the work of overcoming the ravages of tubercle must be left to each country to deal with according to the dictates of its own national genius.

### Tuberculous Infection in the Dog.

A case of canine tuberculosis by direct infection from a human being is reported by G. Guérin in the *Bulletin de la Société de Médecine Veterinaire de Paris.* Six months following the death of its owner from consumption the dog showed signs of breathlessness, cough, expectoration, and loss of flesh, which were sufficiently advanced to necessitate its destruction. At the autopsy tuberculous lesions of the tracheo-bronchial and mediastinal lymphatic glands, as well as of the liver and pericardium, were noticed and verified by microscopic and inoculation methods. Petit of Alfort has already drawn attention to the possibility of canine infection from human tuberculous subjects, and the danger to man from an animal so affected. For a long time past it has been the teaching of the Belgian Veterinary school that canine infection is extremely uncommon in the absence of tuberculous human beings, and that if inquiries be prosecuted with sufficient determination it will be found that an infected animal has had prolonged and intimate contact with a tuberculous individual, so much so, that in doubtful cases in the dog the fact of such a contact having taken place is an important point in settling the diagnosis.

### Sleeping-Sickness.

A considerable amount of evidence has now been accumulated to show that the cases of sleeping-sickness occurring in North-Eastern Rhodesia and Nyasaland have arisen in the absence of *Glossina palpalis*. If this eventually proves to be correct, then some other insect, tsetse fly (*G. morsitans* or *G. fusca*) or otherwise, must be acting as the intermediary or carrier, and this must necessarily render attempts at prophylaxis much more difficult. One explanation is that possibly the Rhodesian trypanosome is a different species with a different host, and some colour is given to this idea by a paper read before the Royal Society by Drs. Stephens and Fantham in November last (1910). They noticed peculiar features in trypanosomes in the blood of rats infected from a patient who had acquired the disease in North-Eastern Rhodesia where *Glossina palpalis* is not found, and after a study of these forms the authors come to the conclusion that they were dealing with a new species of trypanosome and have proposed the name of the *Trypanosoma rhodesiense* for it. More work on different cases from the areas mentioned will be required before one can definitely say if this is so, but even already Dr. Old, of Nyasaland, reports that some of the doctors working there believe that they have also noticed differences from the type species in the trypanosomes of some of their cases. The subject is one of such vast importance that the Government will probably send out a special commission to inquire into the matter on the spot, and until this is done one must suspend judgment as to the peculiarities noted above.

